# Comparative Evaluation of the Effect of Core Type and Antirotational Post on Stress Distribution in an Endodontically Treated Maxillary First Molar: FEA

**DOI:** 10.1155/2022/4336980

**Published:** 2022-05-11

**Authors:** Ramin Mosharraf, Amirhossein Fathi, Seyedeh Shiva Botshekan

**Affiliations:** ^1^Department of Prosthodontics, Dental Materials Research Center, Dental Research Institute, School of Dentistry, Isfahan University of Medical Sciences, Isfahan, Iran; ^2^Dental Students' Research Committee, School of Dentistry, Isfahan University of Medical Sciences, Isfahan, Iran

## Abstract

**Background:**

This study aims to analyze and compare the stress distribution in an endodontically treated maxillary first molar restored with various post and core systems and assess the benefit of the presence of an antirotational post and the effect of its length using finite element analysis.

**Materials and Methods:**

Five 3D models of maxillary first molar restored with variable designs of post and core were constructed using CT scanning and information obtained from textbook and FEM software. Variables were types of core, presence or absence of an antirotational post, and length of the antirotational post. A load of 480 (N) vertically and a load of 240 (N) with 45° to the occlusal plane were applied. Results were analyzed using 3D von Mises criteria.

**Results:**

The results showed that the most homogeneous stress distribution pattern along with dentin and the post-core system was observed in the model with one main post in the palatal canal and in the model with a two-piece core and one main post in the palatal canal and shorter antirotational post in the distal-buccal canal. However, models with one-piece core and antirotational post (either the same length or shorter than the main post) in the distal-buccal canal had the most stress concentration sites.

**Conclusion:**

In this study, it was found that the presence of an antirotational post, its length, and type of core affected the distribution of stress along the dentin and post-core system. This study indicated that if the antirotational post is considered in the post-core design, it is better to use a two-piece core type due to more homogeneous stress distribution along the dentin and post-core system.

## 1. Introduction

The tooth is a complex structure containing pulp, dentin, cementum, and enamel, which is surrounded by periodontal tissue [1]. The natural biomechanical balance of the tooth is adapted to withstand the stress exerted during the chewing process. It is commonly accepted that teeth with severe dental caries or fractures must undergo root canal therapy. Endodontic treatment changes the balance of tooth structure, so restoration of endodontically treated teeth is challenging. One of the most important determinants in the fracture mechanism is the stress distribution pattern along the root. Concentrated stress may initiate and promote crack propagation along weakened surfaces [2]. Post and core systems are commonly used for the restoration of endodontically treated teeth with severe coronal loss. The ability of the post and core system to withstand mastication forces and remain fixed in the tooth is essential for the survival of the restoration [3]. Post and core function is a unit to restore lost tooth structure and provide retention to the crown. The design and selection of the post should reduce the possibility of root fracture from functional forces. Placing post in posterior teeth is challenging due to the anatomy of the root and root canals. It is in agreement that the post, if needed in the posterior region, should be inserted in the largest and straightest canal, namely, the palatal canal in the maxillary molars and distal canal in the mandibular molars [4]. The analysis of stress distribution found in endodontically treated teeth that have been restored with post and core will contribute to identify causes of the high incidence of failure in these teeth. Therefore, it may result in the design of post and core restorations with improved clinical performance.

In last three decades, many authors have investigated the effect of various factors on the stress distribution using the finite element analysis, and factors include postmaterial, ferrule height, and different designs. Upadhyaya et al. [5] concluded that tapered post produced greater stresses than parallel posts, irrespective of other parameters such as presence of ferrule or type of material. Similar findings were found in studies conducted by Peter et al. [6]. The material of dental posts and presence or absence of ferrule are the factors affecting the stress distribution. Several studies have evaluated the effect of post materials and presence or absence of ferrule. A number of studies have reported the cervical region and the area between the middle and cervical thirds of the root as a stress concentration site [5, 7]. Presence of ferrule definitely reduces the stress in cervical area and has the most uniform pattern of stress distribution [5]. Aykent et al.[8] reported that presence of 1 mm of coronal dentin above the shoulder significantly increased the fracture strength of teeth restored with the post and core system. Icim et al. [9] in their FEM study reported that when ferrule height reached 1.5 mm, displacement and rotation of crown reduced. About post materials, some studies reported that models with metal-ceramic restorations showed greater stresses in the cervical region and the metallic cast posts showed the slightest stress concentration. Stainless steel, Ti, and ceramic posts have favorable stress distribution in comparison with FRC posts, and FRC posts showed the higher stress level in the area between the middle and cervical thirds of the root [5, 10–13]. Rita Eid et al. claimed that there are no statistically significant differences in the fracture resistance between endodontically treated teeth with post and cores made of different CAD/CAM materials in comparison with cast post and cores [14].

Post and core design is one of the most important factors in failure of restoration. There are different designs for the manufacturing of dental post and cores, and the ideal one would provide retention without extra stresses. One important design consideration in post and core is antirotation. Antirotation can be incorporated in the post and core with extra post, slots, or pins. When an antirotational post is needed, the core can be fabricated in one piece or two pieces. This study is designed to evaluate the effect of antirotational post presence, its length, and type of core in the stress distribution pattern. However, it is difficult to directly measure the stress applied to the root. Finite element analysis is a numerical method to investigate stress and strain in any complex system. This method allows the calculation of stresses, strains, and deformations in an arbitrarily shaped 3D finite element model representing a structure under static loading. It has recently become a powerful technique in dental biomechanics. It is considered to be fast, precise, and dependable alternative compared to in vivo and in vitro investigation methods [15].

## 2. Materials and Methods

The finite element analysis system is adopted to model and analyze the stress accumulation in the teeth restored with post and core. Before attempting to prepare any canal space for post installation, it is important to know the anatomy of maxillary 1^st^ molar root to avoid root perforation.

**Figure 1:**
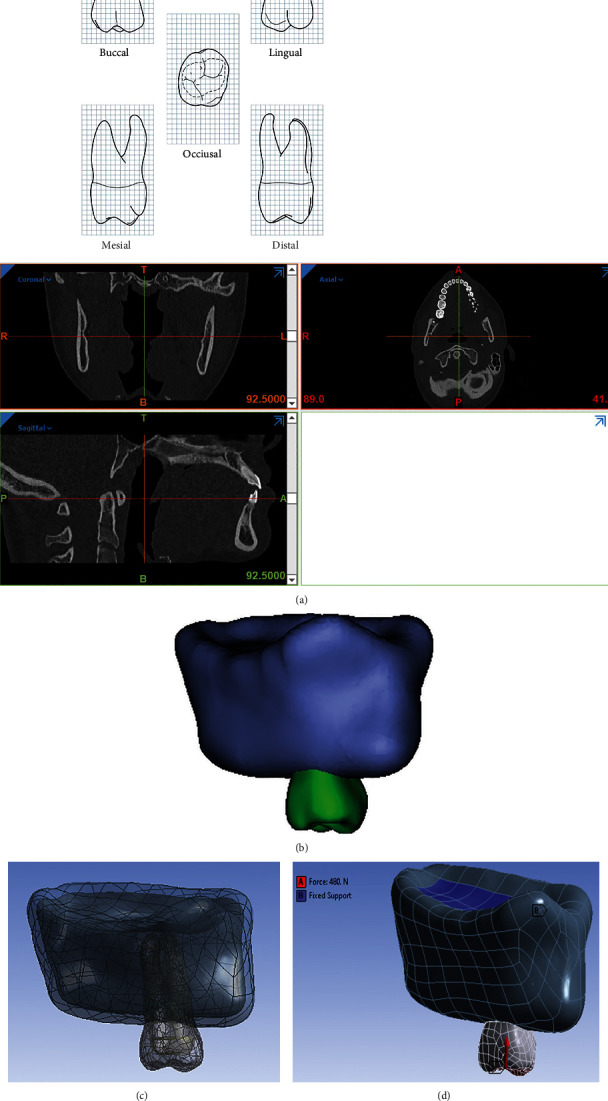
(a) Maxillary right first molar (grid = 1 sq.·mm) CT scan images. (b) Model in STL format. (c) Model in STP format. (d) Application of vertical and oblique forces.

A 3D model of maxillary 1st molar is simulated based on the information obtained from Wheelers's dental anatomy, physiology, and occlusion [16]. External shapes of the roots were generated based on Figure 1(a)from textbook [16]. Models of bone, molar teeth, post, and core are created in 3-Matic and Mimics software. First, CT scan images with a distance of 1 mm between the slices were entered into Mimics software (Figure 1(a)). Using segmentation tools, masks for maxilla teeth and posts were created, and then, 3D models of these components were created using the calculate 3D command (Figure 1(b)). Then, all the parts are exported from software with STL format. Then, these parts change to STP format in Geomagic software (Figure 1(c)). After converting all geometries to STP format, these geometries were entered into ANSYS software for analysis. Finally, the forces intended to mimic the function of the tooth are applied (Figure 1(d)). Stress distribution in FEM analysis is generally defined as von Mises stress which could be maximum and minimum principal stress or it could be principal strain. The von Mises stress is evaluated in three planes, which are *x*-axis, *y*-axis, and *z*-axis using a formula.

In this study, the 3D meshes totally generated 58781 tetrahedral elements and 118519 nodes. The model was subsequently subjected to access opening, root canal shaping, and filling, simulating the clinical process [2]. All three canals were shaped with 0.06 taper instruments. The canal was filled using gutta percha. Posts were tapered and conformed shapes of canals. To insert the post, either the palatal or distal-buccal canal was subjected to drilling of ideal angulation and depth. A post of 1.1 mm in diameter in cervical part and 9.0 mm in length was inserted into the palatal canal, and a post of 1.1 mm in diameter in the cervical region and 4.0 mm or 9.0 mm in length was inserted into the distal-buccal canal as antirotational post. Posts were cemented with 0.1 mm layer of Panavia [2]. To simulate a tooth with severe loss of coronal structure, most of the coronal component was removed, so only 2.0 mm ferrule remained; the removed structures were replaced with the crown. We consider 2.0 mm ferrule because presence of adequate ferrule has a positive effect on fracture resistance of endodontically treated teeth [16]. Different designs of post and core were used in modeling: one-piece core with the main post in the palatal canal, one-piece core with the main post in the palatal canal and isometric antirotational post in the distal-buccal canal, one-piece core with the main post in the palatal canal and shorter antirotational post in the distal-buccal canal, two-piece core with the main post in the palatal canal and shorter antirotational post in the distal-buccal canal, and two-piece core with the main post in the palatal canal and isometric antirotational post in the distal-buccal canal. Cr-Co was chosen as the material for post and core. The finish line of the tooth was chamfer. The tooth material was assumed to be isotopic, homogeneous, and elastic. The bone supporting the root was assumed to be rigid. The elastic moduli and Poisson's ratio of the materials used in this study are taken from previous articles, as given in Table 1 [2, 7]. An external load of 480 (N) vertically to mimicking intercuspal occlusion was applied vertically to the occlusal surface, and a load of 240 (N) with 45° angle to the long axis of the tooth was applied on the occlusal surface of tooth simulating mastication [17]. The models were analyzed by Ansys 2020 software, and applied stress to the component was measured and converted into color graphics.

## 3. Results

**Figure 2:**
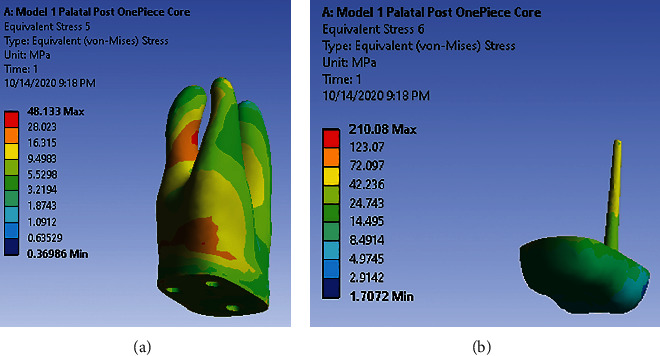
(a) Stress distribution pattern in dentin under 480 (N) along model 1. (b) Stress distribution pattern in post-core under 480 (N) along model 1.

**Figure 3:**
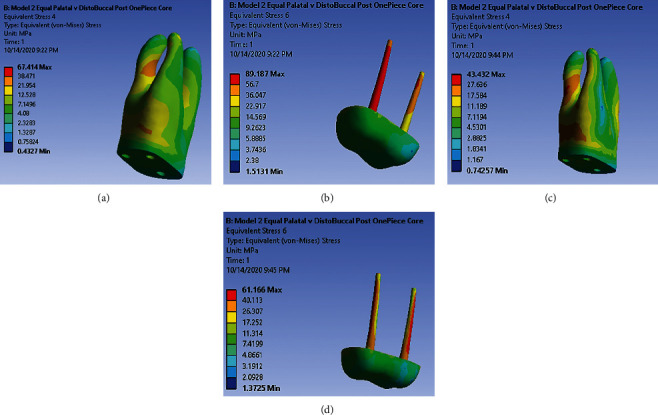
(a) Stress distribution pattern in dentin under 480 (N) along model 2. (b) Stress distribution pattern in post-core under 480 (N) along model 2. (c) Stress distribution pattern in dentin under 240 (N) along model 2. (d) Stress distribution pattern in post-core under 240 (N) along model 2.

**Figure 4:**
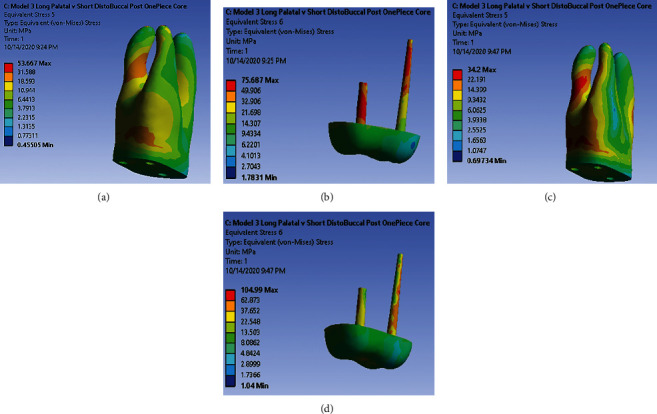
(a) Stress distribution pattern in dentin under 480 (N) along model 3. (b) Stress distribution pattern in post-core under 480 (N) along model 3. (c) Stress distribution pattern in dentin under 240 (N) along model 3. (d) Stress distribution pattern in post-core under 240 (N) along model 3.

**Figure 5:**
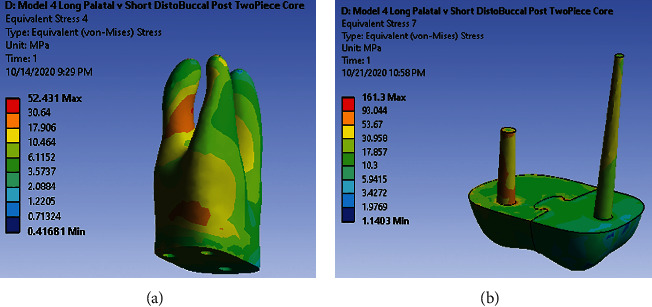
(a) Stress distribution pattern in dentin under 480 (N) along model 4. (b) Stress distribution pattern in post-core under 480 (N) along model 4.

**Figure 6:**
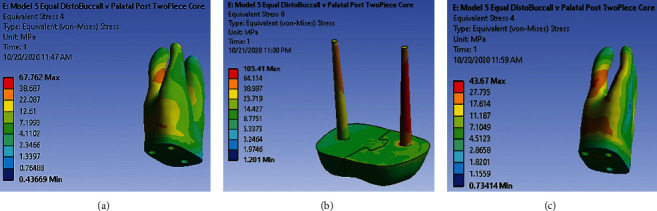
(a) Stress distribution pattern in dentin under 480 (N) along model 5. (b) Stress distribution pattern in post-core under 480 (N) along model 5. (c) Stress distribution pattern in dentin under 240 (N) along model 5.

**Figure 7:**
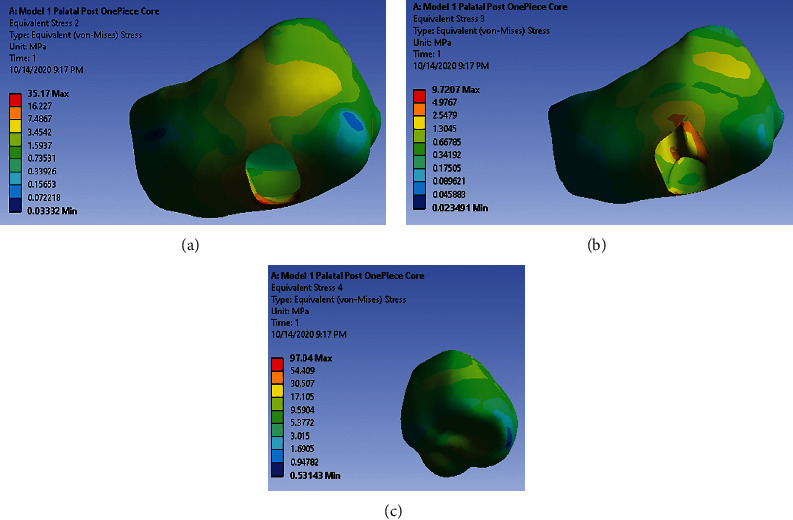
(a) Stress distribution pattern in cortical bone. (b) Stress distribution pattern in spongy bone. (c) Stress distribution pattern in crown.

The stress distribution under the influence of these forces was evaluated separately in the cortical bone, spongy bone, crown, dentin, and post-core system. The root canals were divided into three portions: the coronal third, middle third, and apical third. Figures 2–7 show the equivalent von Mises stress distribution in each model. To make it easier to compare the stress distribution in the 5 models, areas in red were considered as very high, in orange as high, in yellow as a medium, and others as low-stress concentration sites.

### 3.1. Model 1: One-Piece Core with the Post in the Palatal Canal

Under loading 480 (N) vertically, dentin in the middle third of the distal-buccal root shows very high and high-stress concentration. Root trunk in buccal shows high-stress concentration (Figure 2(a)). There is no high-stress concentration site in the palatal post and one-piece core (Figure 2(b)). Under loading 240 (N) with 45°angle to the long axis of the tooth, dentin and the post-core system had low-stress concentrations in all sites.

### 3.2. Model 2: One-Piece Core with the Main Post in Palatal and Isometric Antirotational Post in the Distal-Buccal Canal

Under loading 480 (N) vertically, dentin in the middle third of the distal-buccal root shows high-stress concentration (Figure 3(a)). In two-third apically part of the palatal post, high-stress accumulation is observed. All length of antirotational posts in the distal-buccal canal shows very high and high-stress concentrations (Figure 3(b)). Under loading 240 (N) with 45° to the long axis of the tooth, high-stress accumulation is observed in the dentin of the coronal third and middle third of the distal-buccal root (Figure 3(c)). Very high and high-stress accumulation is observed throughout the length of both main and antirotational posts (Figure 3(d)).

### 3.3. Model 3: One-Piece Core with Main Palatal Post and Short Antirotational Post in the Distal-Buccal Canal

Under 480 (N) force vertically, high-stress concentration is observed in dentin of the middle third of the distal-buccal root and a small region in the root trunk (Figure 4(a)). Very high and high-stress accumulation is observed, throughout the whole length of antirotational post and two-thirds apical part of main post (Figure 4(b)). Under loading 240 (N) with 45° to the long axis of tooth, very high and high-stress concentration is observed in dentin of coronal third and middle third of distal-buccal root and large part of root trunk in the buccal side (Figure 4(c)). High-stress concentration is also seen in dentin of coronal and middle third of mesial-buccal root. There is no very high-stress accumulation in the post-core system in this model. High-stress concentration is observed throughout the length of the palatal post and a small region in antirotational post (Figure 4(d)).

### 3.4. Model 4: Two-Piece Core with the Main Post in Palatal and Shorter Antirotational Post in Distal-Buccal Canal

Under loading 480 (N) vertically, dentin in the middle third of distal-buccal root is the stress concentration site. Also, in small region of the root trunk, high-stress is observed (Figure 5(a)). There are scattered areas with high-stress concentration in antirotational post (Figure 5(b)). Under loading 240 (N) with the angle of 45° to the long axis of the tooth, no very high and high-stress concentration areas along dentin and post and core were observed.

### 3.5. Model 5: Two-Piece Core with Palatal Post and Isometric Antirotational Post in Distal-Buccal Canal

Under loading 480 (N) vertically, high-stress accumulation is observed in the middle third of distal-buccal root dentin (Figure 6(a)). Very high and high-stress accumulation is observed throughout the palatal post. The apical half of the antirotational post is the site of high-stress accumulation (Figure 6(b)). Under loading 240 (N) with the angle of 45° to the long axis of tooth, dentin is in the coronal third and middle third of distal-buccal root. Root trunk and two-third coronal of mesial-buccal canal are the sites of high-stress accumulation (Figure 6(c)). There is no high and very high-stress concentration site along the post-core.

The results show that the direction of the applied force, type of core, the presence or absence of antirotation post, and its length all affect the pattern of stress distribution and stress concentration in the dentin and post and core system. However, they did not have significant influence on stress distribution in bone and crown. Figures 7(a)–7(c) are examples of stress distribution pattern in bone and crown.

## 4. Discussion

The present biomechanical study was performed to evaluate the importance of the presence of antirotational post and its feature and type of core in the stress distribution of maxillary first molar. Since no previous study had examined the role of these two in the distribution of stress in maxillary molar, this study evaluated the issue in this area. It is accepted that posts should be used only for retaining the core, not with intention of reinforcing an endodontically treated tooth. The most common failures in post and core systems are loosening of the post and tooth fracture. Posterior teeth are subjected to greater stresses due to being closer to the transverse horizontal axis than anterior teeth. This combined with the morphologic characteristic makes them more vulnerable to fracture [17]. Endodontic procedures and post drilling can cause damage, especially microcracks on the surface of root canals. Therefore, stress concentration may cause crack propagation and fracture along these weakened surfaces [2]. An ideal post should diffuse the functional stresses along the root surface in such a manner that minimum stresses are developed [17]. Teeth are restored with post and core subjected to various types of forces. Torsional forces on the post-core unit may cause the post to loosen and displace, leading to the failure of the system. Therefore, the resistance of the post-core system will play a key role in stabilizing and retaining [18]. Resistance means the ability of the post and tooth to sustain lateral and rotational forces. It is influenced by the remaining tooth structure, post's length, rigidity, the presence of antirotation features, and the presence of a ferrule. Richard S et al. claimed that the presence of antirotational features affects the post and core resistance [19]. On the contrary, Rapso LHA et al. concluded that the presence and location of antirotational devices does not affect fracture resistance. However, antirotational features influenced the stress distribution within the tooth structure [20]. The antirotational feature should provide resistance to torsional forces but should not elevate the fracture failure rate. It is essential to preserve as much tooth structure as possible, especially in the root canal. To conserve intact dental structure, antirotation post should only be considered when necessary. In the final analysis, the quality of the root canal thereby combined with the quality of its final restoration determines the clinical success of pulpless tooth with post and core [19]. The use of post and core material with a lower elastic modulus and cement with a higher elastic modulus reduces cement and post and core deformation and leads to reduced post and core stress. Therefore, the Ni-Cr metal ceramic crown/Au-Pd post and core/Panavia may be desirable for post and core restorations [21].

In this study, we investigated the effect of extra posts on the role of antirotation and the type of core in stress distribution by FEA. In a two-piece post and core, the difference in a path of insertion can provide sufficient retention for the core [4, 22]. From the obtained results, it is inferred that the stress distribution is related to the angle of applied force, the length of the post, and the kind of core (one or two pieces). Eshelman E et al. reported that the angle of the load affects the amount of force required to create the fracture and its location [19]. Burns DA et al. concluded that when the force is applied obliquely, larger diameter posts at increased depth accumulate stress more efficiently than smaller shorter posts [19]. Hunter A et al. concluded that post length is more important than post diameter in stress concentration in the cervical region [23]. However, short, wide posts led to elevated stress concentration in this region [24]. Overall, this study showed that models 1 and 4 were the best post and core kinds for homogeneous stress distribution, while the worst models that led to high-stress accumulation in dentin and post were models 2 and 3. The FEA model can be applied to various physical problems and its power lies in its versatility. FEA outcomes give complete geometry of post and core and tooth, material properties, loading conditions, and stress distribution pattern along with the tooth and post and core. Advantages of FEM are as follows: it enables the visualization of superimposed structures, and researcher can locate the magnitude and direction of an applied force, it provides stress points that can be measured theoretically, it is easy to repeat, and it is a noninvasive technique [25].

The results of our study are in line with the results of the Burns DA et al.' study when the core is one-piece. That is, when the length of the antirotational post is longer, the pattern of stress distribution is more uniform. However, in the case of a two-piece core, the results contradict the results of the Burns DA et al.' study, which can be due to the difference in the path of insertion of the two posts, while the core is two-piece, and the other is that the posts are prefabricated in Burns DA et al.' study [19]. Comparing Tables 2 and 3, we find that the maximum stress applied to dentin in both the oblique and vertical forces is not much different, although the force applied in the vertical position is twice that of the force applied in the oblique position, so it is in line with Eshelman E et al.' study [19] which states the angle of force affects the maximum stress required to cause failure. The results of this study agree with Raposo et al.' [25] study in which antirotational feature affects the pattern of stress distribution.

In this study for simplicity and utility on finite element analysis, all materials were presumed to be homogeneous and isotropic; however, this may not match clinical reality. Another simplification done in this study is not considering the effect of periodontal ligament, assuming that it will not have much effect on the final result. More in vivo studies on the current topic are therefore recommended.

## 5. Conclusions

Based on the result of this study,

1. Presence of antirotational post and its properties affect the distribution of stress in the tooth
2. It is recommended to fabricate a two-piece core when an antirotational post is considered in the post-core design.
3. When the core is one-piece, it is better not to use antirotational post and fabricate the post-core with only main post.

## Figures and Tables

**Table 1:** Material properties.

Material	Elastic modulus (GPA)	Poisson's ratio
Enamel	84.10	0.33
Dentin	18.60	0.31
Pulp	0.0068	0.45
Periodontal ligament (0.2 mm thickness)	0.07	0.45
Gutta percha	0.07	0.40
Adhesive resin cement (Panavia)	18.6	0.31
Cortical bone/cancelleous bone	13.7/1.37	0.3
Metal cast post (Ni-Cr)	188	0.33

**Table 2:** Maximum values of von Mises stress (MPa) in the models with vertically 480 (N) loading.

Model	Dentin	Main post	Antirotational post	Cortical bone	Spongy bone	Crown
1	48.133	72.097	—	35.17	9.720	17.105
2	38.471	89.187	89.187	36.835	5.2135	16.734
3	31.588	75.658	75.687	37.045	5.1771	16.363
4	30.64	53.67	93.044	35.887	5.0599	18.053
5	38.687	105.41	64.114	36.809	5.2124	16.669

**Table 3:** Maximum values of von Mises stress (MPa) in the models with 240 (N) obliquely loading.

Model	Dentin	Main post	Antirotational post	Cortical bone	Spongy bone	Crown
1	37.047	44.777	—	27.1	5.4831	12.758
2	43.432	61.166	61.166	29.268	6.091	22.481
3	34.2	62.873	62.873	29.308	6.0733	12.9
4	29.299	48.979	95.411	28.01	5.7208	11.761
5	43.67	68.492	68.492	29.236	6.0818	12.479

## Data Availability

The data used to support the findings of this study are available from the corresponding author upon request.
